# Assessing the Impact of Gender and COPD on the Incidence and Mortality of Hospital-Acquired Pneumonia. A Retrospective Cohort Study Using the Spanish National Discharge Database (2016–2019)

**DOI:** 10.3390/jcm10225453

**Published:** 2021-11-22

**Authors:** Javier de-Miguel-Diez, Rodrigo Jimenez-Garcia, Valentin Hernandez-Barrera, Jose M. de-Miguel-Yanes, David Carabantes-Alarcon, Ana Lopez-de-Andres

**Affiliations:** 1 Respiratory Department, Instituto de Investigación Sanitaria Gregorio Marañón (IiSGM), Hospital General Gregorio Marañón, Universidad Complutense de Madrid, 28040 Madrid, Spain; javier.miguel@salud.madrid.org; 2 Department of Public Health & Maternal and Child Health, Faculty of Medicine, Universidad Complutense de Madrid, 28040 Madrid, Spain; dcaraban@ucm.es (D.C.-A.); anailo04@ucm.es (A.L.-d.-A.); 3 Preventive Medicine and Public Health Teaching and Research Unit, Health Sciences Faculty, Universidad Rey Juan Carlos, Alcorcón, 28922 Madrid, Spain; valentin.hernandez@urjc.es; 4 Internal Medicine Department, Instituto de Investigación Sanitaria Gregorio Marañón (IiSGM), Hospital General Gregorio Marañón, Universidad Complutense de Madrid, 28040 Madrid, Spain; josemaria.demiguel@salud.madrid.org

**Keywords:** hospital-acquired pneumonia, ventilator-associated pneumonia, non-ventilator hospital-acquired pneumonia, COPD, gender, incidence

## Abstract

Background: We aim to analyze incidence and outcomes of patients hospitalized with hospital-acquired pneumonia (HAP) according to chronic obstructive pulmonary disease (COPD) status and sex in Spain (2016–2019). Methods: We conducted a retrospective cohort study using national hospital discharge data of patients ≥40 years with a primary diagnosis of HAP, using the specific diagnostics of non-ventilator (NV)-HAP and ventilator-associated pneumonia (VAP). Results: We identified 37,029 patients with HAP ((NV)-HAP 87.28%, VAP 12.72%), 13.40% with COPD. HAP incidence increased over time, but only in subjects without COPD (*p* < 0.001). In women, incidence of HAP and (NV)-HAP was similar regardless of COPD status, but VAP incidence was lower in COPD women (*p* = 0.007). In men, the incidence of (NV)-HAP was significantly higher in those with COPD, while VAP incidence was lower in COPD men (*p* < 0.001). The in-hospital mortality (IHM) was similar in men and women with and without COPD. The risk of dying in hospital increased with age, congestive heart failure, cancer, and dialysis among men and women with COPD. Men that underwent surgery had a lower risk of IHM. VAP increased 2.58-times the probability of dying in men and women. Finally, sex was not associated with IHM among COPD patients. Conclusions: Incidence of HAP was significantly higher in COPD patients than in those without COPD, at the expense of (NV)-HAP but not of VAP. When stratifying by sex, we found that the difference was caused by men. IHM was similar in COPD and non-COPD patients, with no significant change overtime. In addition, sex was not associated with IHM.

## 1. Introduction

Hospital-acquired pneumonia (HAP) is a severe nosocomial infection that affects many hospitalized patients and is associated with increased morbidity, mortality, and health costs [1]. Most epidemiologic and etiologic studies in this field have been focused on critically ill patients [2]. In this regard, ventilator-associated pneumonia (VAP) is a subgroup of HAP that occurs in mechanically ventilated patients more than 48 h after tracheal intubation [3]. Another subcategory of HAP is non-ventilator HAP ((NV)-HAP). Although both (NV)-HAP and VAP cause considerable clinical and economic burdens, most of the published studies focuses primarily on VAP [4]. This is probably the result of the greater severity of this disease in patients in the intensive care unit (ICU) setting, as well as the ability to more precisely define the presence of true infection in mechanically ventilated patients with pneumonia using diagnostic techniques such as bronchoalveolar lavage (BAL) with quantitative cultures [5]. 

HAP often has an unfavorable prognosis, particularly among older hospitalized patients or those with chronic comorbidities [6]. These diseases can lead to immune suppression, causing impairments of vital organs such as lungs, making the patient more vulnerable to infection [7]. Nonetheless, the relation between HAP and chronic obstructive pulmonary disease (COPD) has not been thoroughly analyzed in the literature until now. Although it has been described that COPD could be an independent predictor of VAP [8], there are hardly any studies on (NV)-HAP and COPD [9].

Available studies suggest that there may be gender-related differences in HAP outcomes. Thus, the incidence might be higher among men [10] while women could have a higher mortality [11], although there are discordant data in this regard [12]. However, there are no studies in the literature aimed at evaluating the influence of COPD on this relationship. The availability of these data could influence hospitals and physicians to increase efforts aimed at preventing HAP in men and women with COPD, as well as improving the treatment of this nosocomial infection in such patients.

We analyzed the incidence, clinical characteristics, use of therapeutic procedures, and in-hospital outcomes in patients hospitalized with HAP according to COPD and sex. We used propensity score matching (PSM) to compare in-hospital outcomes after HAP between men and women with and without COPD, and between men and women with COPD. Finally, we identified the variables associated with in-hospital mortality (IHM) for patients with COPD according to sex. 

## 2. Materials and Methods

We have conducted a retrospective cohort study using the Spanish Register of Specialized Care-Basic Minimum Database (RAE-CMBD, Registro de Actividad de Atención Especializada-Conjunto Mínimo Básico de Datos) for the period between 1 January 2016 and 31 December 2019. The discharge records are coded based on the International Classification of Disease, Tenth Revision (ICD-10). More details on RAE-CMBD are available online (accessed on 22 October 2021) [13].

We selected patients aged ≥40 years with a primary diagnosis of HAP, using the specific diagnosis of (NV)-HAP or VAP, as indicated in Table S1. As can be seen in this table only patients with a “Not present at admission” indicator coded who had a hospitalization for 48 h or more were considered to suffer a HAP.

The population was divided according to sex and to the presence of COPD. Subjects with a diagnosis code for COPD (J44.0, J44.1, and J44.9) in any diagnosis field were classified as having COPD. 

The main study variables were trends in the incidence of HAP, (NV)-HAP and VAP among men and women with and without COPD. Incidences were calculated based on the Spanish hospitalized population with and without COPD grouped by age group and sex according to the RAE-CMBD data [14].

Other study variables analyzed included age, comorbidities, therapeutic procedures, IHM, and length of hospital stay (LOHS) of patients with HAP. 

Comorbidity was quantified using the Charlson Comorbidity Index (CCI) calculated based on ICD-10 codes, as described elsewhere [15]. 

The RAE-CMBD includes a variable with the Diagnosis-Related Groups categorized as “Medical/Surgical/Other” that was used to identify patients who underwent any type of surgical procedure during their hospital admission [13].

Concerning procedures, we studied bronchial fibroscopy, computerized axial tomography of thorax, dialysis, and oxygen prior to hospitalization (see ICD-10 codes in Table S1). Finally, we also analyzed the presence of bronchiectasis as comorbidity.

Regarding the isolation of pathogens among patients with pneumonia, we only identified those coded that were confirmed by laboratory, including *Aspergillus*, *Candidiasis*, *Escherichia coli*, *Haemophilus influenzae*, *Klebsiella pneumoniae*, *Legionella*, non-specified Streptococcus, other Gram-negative bacteria, *Pseudomonas aeruginosa*, *Staphylococcus aureus*, *Streptococcus pneumonia*, *Influenza virus*, and another virus (Table S1).

We have used a PSM method to create subpopulations that were comparable based on their baseline conditions [16]. We have performed three PSM analyses, namely, women with COPD and non-COPD women, men with COPD and non-COPD men, and COPD men and COPD women. The PSM was conducted using multivariable logistic regression in which the matching variables were age, sex and comorbid conditions present at admission. 

### 2.1. Statistical Analysis

Incidences were analyzed using Poisson regression models adjusted for age and sex when required, providing incidence rate ratios (IRR) with 95% confidence intervals (95%CI). 

Descriptive statistical analysis included mean and standard deviation (SD) or median and interquartile range (IQR) for continuous variables and frequency and percentage for categorical variables.

Continuous variables were compared using the t test or Mann–Whitney test. Categorical variables were compared using the chi-square test.

McNemar’s test and a paired *t* test were used to compare study subgroups after PSM [17].

To identify which variables were independently associated with IHM we conducted multivariable logistic regression. We constructed models separately for men and women and according to COPD status. Finally, using the entire database of patients with COPD, we analyzed the effect of sex. The results are shown as odds ratios (ORs) with their 95% CIs.

The statistical analysis and PSM were conducted using Stata version 14 (Stata, College Station, TX, USA), and significance was set at *p* < 0.05 (2-sided).

### 2.2. Ethics

The RAE-CMBD is owned by the Spanish Ministry of Health and can be accessed upon request [18]. Given the characteristics of this registry, which is anonymous, it does not require individual written consent from the patients or ethics committee approval according to the Spanish legislation.

## 3. Results

A total of 37,029 patients (65.39% men and 34.61% women) aged ≥40 years were hospitalized with a diagnosis of HAP in Spain during the period 2016–2019. (NV)-HAP diagnosis was identified in 32,319 patients (87.28%) and VAP diagnosis in 4710 patients (12.72%). COPD was diagnosed in 9924 patients (13.40%) with HAP. The prevalence of COPD was higher among men than among women (17.29% vs. 6.04%; *p* < 0.001) with HAP.

Table 1 show the incidence, clinical characteristics and in-hospital outcomes of patients admitted to hospitals with a diagnosis of HAP according to COPD status. We have found that the incidence of HAP only increased in subjects without COPD (250 cases per 100,000 hospitalized subjects without COPD in 2016 to 288 in 2019; *p* < 0.001), both in those with (NV)-HAP (217 in 2016 to 246 in 2019; *p* < 0.001) and those with VAP (33 in 2016 to 42 in 2019; *p* < 0.001). However, in subjects with COPD, the incidence of HAP, (NV)-HAP and VAP remains stable throughout the study period.

Incidence was significantly higher in people with COPD than in non-COPD people for HAP and (NV)-HAP and for all years analyzed (*p* < 0.001). However, the incidence of VAP was higher in non-COPD subjects over the study period.

Using the Poisson regression model, we found that the incidence of HAP was 1.10-times higher among patients with COPD than among those without COPD (IRR 1.10; 95%CI 1.08–1.12). When we stratified by pneumonia type, the incidence of (NV)-HAP resulted in an IRR of 1.18 (95%CI 1.14–1.22) and for VAP a not significant IRR of 0.87 (95% 0.73–1.02).

Mean age and mean CCI increased significantly over time only in patients without COPD. LOHS was around 22 and 24 days in patients with and without COPD, respectively. IHM was around 30% in COPD patients and around 29% in non-COPD patients with no significant change overtime.

Influenza virus increased significantly over time only in patients without COPD (*p* < 0.001). *Pseudomonas aeruginosa* decreased significantly only in patients with COPD (6.08% vs. 5.36%; *p* = 0.021) and non-specified Streptococcus decreased only in non-COPD patients (0.57% vs. 0.45%; *p* = 0.044), as can been seen in Table S2.

Table 2 and Table 3 show clinical characteristics and in-hospital outcomes for women and men admitted to hospitals with a diagnosis of HAP according to the presence of COPD.

In women with and without COPD, the incidence of HAP and (NV)-HAP overtime was similar and around 200 and 190 cases per 100,000 hospitalized women, respectively. However, for VAP, the incidence was significantly lower in women with COPD (15 cases per 100,000 women hospitalized with COPD vs. 22 cases per 100,000 women hospitalized without COPD, *p* = 0.007) (Table 2).

Mean age was around 74 years in both women with and without COPD. Although the mean CCI was 1.42 in both groups, women without COPD had a higher prevalence of cerebrovascular disease, dementia, hemiplegia, or paraplegia and cancer/metastatic cancer. However, prevalence of myocardial infarction, congestive heart failure and AIDS was significantly higher in women with COPD. No differences were observed for bronchiectasis with low prevalence’s in both groups of women.

During hospitalization, women with COPD received dialysis and underwent surgery significantly less often than women without COPD (*p* = 0.034 and *p* = 0.001, respectively); however, women with COPD received more frequently oxygen prior to hospitalization (11.87% vs. 2.2%; *p* < 0.001). The mean LOHS was significantly lower in women with COPD than in non-COPD women (21 days vs. 23 days; *p* = 0.016). The crude IHM was around 27% in both groups.

As can been seen in Table 2, after PSM, the use of therapeutic procedures in both groups remain similar as before PSM. The IHM was 27.65% in COPD women and 30.32% in non-COPD women (*p* = 0.239).

In men with and without COPD, the crude incidences of HAP were 368 and 350 cases per 100,000 hospitalized men (*p* < 0.001). The incidence of (NV)-HAP was significantly higher in men with COPD (340 cases per 100,000 men hospitalized with COPD vs. 297 cases per 100,000 men hospitalized without COPD, *p* < 0.001). However, the incidence of VAP was significantly lower in men with COPD (28 cases per 100,000 men hospitalized with COPD vs. 52 cases per 100,000 men hospitalized without COPD, *p* < 0.001) (Table 3). 

Before PSM, we found that men with COPD were significantly older (75.39 years old vs. 69.95 years old; *p* < 0.001) and had higher mean of CCI. Specifically, men with COPD had higher prevalence of congestive heart failure, peripheral vascular disease, type 2 diabetes mellitus and renal disease; however, they had lower prevalence of cerebrovascular disease, hemiplegia or paraplegia and cancer/metastatic cancer. As reported for women, no differences were found for bronchiectasis.

As can been seen in Table 3, men with COPD received less bronchial fibroscopy, dialysis, and undergone surgery (all *p* < 0.001); however, the prevalence of oxygen prior to hospitalization is significantly higher in men with COPD (9.03% vs. 1.59%; *p* < 0.001). LOHS was lower in men with COPD (22 days vs. 25 days; *p* < 0.001). IHM was around 30% in both men with and without COPD.

After PSM, prevalence of use of computerized axial tomography of thorax, dialysis, undergone surgery, and the hospital stay was significantly higher in non-COPD men (all *p* < 0.001). The prevalence of oxygen prior to hospitalization remains significantly higher in patients with COPD (*p* < 0.001). There were no changes regarding the IHM (Table 3).

Table 4 show the incidence, clinical characteristics and hospital outcomes for COPD patients admitted to hospital with a diagnosis of HAP according to sex.

The crude incidences of HAP, (NV)-HAP, and VAP were significantly higher in men than in women with COPD (all *p* < 0.001). The results of the Poisson regression model showed that the incidence of HAP, (NV)-HAP, and VAP during the period 2016–2019 was higher in COPD men than COPD women (for HAP: 1.61; 95%CI 1.50–1.72; for (NV)-HAP: 1.69; 95%CI 1.53–1.82; for VAP: 1.79; 95%CI 1.35–2.40).

When we compare COPD men with COPD women, we observe that men were older (75.39 ± 10.19 years old vs. 74 ± 11.97 years old; *p* < 0.001), with a higher mean CCI (1.67 ± 1.17 vs. 1.42 ± 1.15, *p* < 0.001). Men also more frequently had peripheral vascular disease, T2DM, liver disease (mild and moderate/severe), renal disease, and cancer/metastatic cancer. However, congestive heart failure and rheumatoid disease were more prevalent in women than in men. 

Before PSM, men received more frequently dialysis (5.49% vs. 3.35%; *p* = 0.013). Furthermore, prevalence of surgery was higher in men (43.68% vs. 38.45%; *p* = 0.007). However, men received less frequently oxygen prior to hospitalization (9.03% vs. 11.87%; *p* = 0.012). IHM were 31.19% in COPD men and 27.61% in COPD women (*p* = 0.047).

After PSM, no significant differences were found between men and women regarding the use of therapeutic procedures (except the prevalence of undergone surgery which is still significantly higher in men; *p* = 0.020), LOHS and IHM.

Regarding the pathogen’s isolation, after PSM, the distribution of pneumonia pathogens was similar between men and women with COPD, as can been seen in Table S3.

Table 5 show the multivariable analysis of variables associated with IHM among COPD men and women with HAP. The risk of dying in hospital increased with age, congestive heart failure, and cancer/metastatic cancer among men and women with COPD. However, hemiplegia or paraplegia (OR 1.66; 95%CI 1.21–2.3) was a factor associated with IHM only in men.

Among men those who underwent surgery had lower risk of IHM (OR 0.71; 95%CI 0.61–0.81). However, the need for dialysis during admission increased the risk of IHM in COPD patients irrespective of sex (OR 4.11; 95%CI 2.4–7.05).

When possible, confounders were controlled, VAP increased 2.58-times the probability of dying in both men and women (OR 2.58; 95%CI 1.66–4.00). In either group, men and women, no association was found for year of admission. Finally, as found with the PSM, sex was not associated with IHM (men: OR 1.09; 95%CI 0.87–1.38).

## 4. Discussion

The current study, to the best of our knowledge, is the first to explore in detail the relationship between HAP and COPD according to sex. We found that the incidence of HAP was significantly higher in people with COPD than in people without COPD, but this was due to (NV)-HAP, since the incidence of VAP was higher in subjects without COPD during the study period. When stratifying by sex, we also detected that the difference was caused by men, since the differences were not significant in women, probably due to their lower number. On the other hand, the incidence of VAP was lower in both men and women with COPD compared to those without COPD. 

In line with our results, previous studies have shown that COPD is a risk factor for HAP [2]. It has also been described that patient who develop (NV)-HAP have more comorbidities including COPD [5,19]. The greater susceptibility of COPD patients to the development of pneumonia may be due to their clinical characteristics, such as mucus production in patients with chronic bronchitis and the potential presence of pathogenic bacteria in the airways, which may increase during exacerbations and be associated with increased inflammation and the host immune response [20]. In addition, abdominal opening or inflation (in endoscopic surgeries) alter chest wall configuration and may cause mechanical dysfunction in COPD, predisposing to infection [21,22,23]. However, in our study it is striking that incidence of VAP was lower in both men and women with COPD compared to those without COPD, contradicting a number of earlier studies that identified COPD as an independent risk factor for development of VAP [24,25,26]. In the same way, Koulenti et al. [27] found that incidence of VAP was not different in patients with and without COPD. In addition, a large prospective observational study found no significant difference in the incidence of VAP between non-exacerbated COPD patients and those without COPD. Probably, the most severe COPD cases do not meet safety requirements to undergone surgery. In addition, it is possible that patients are less likely to be intubated if they have COPD than if they do not have COPD [28]. In fact, current investigation suggests that non-invasive ventilation can be used as an accessory to treatment for preventing intubation in patients with a severe COPD exacerbation, lowering the risk of death, and halting treatment failure [29].

Contrary to what has been described in other studies [19], we found that incidence of HAP increased in subjects without COPD over time, both in those with (NV)-HAP and those with VAP. However, in subjects with COPD, incidence figures remain stable throughout the study period. The absence of an increase in the incidence in COPD patients over time could be due to the development of quality improvement programs focused on HAP in this subgroup of patients, although the characteristics of our study do not allow us to clarify this question. Further robust evaluation of facility level exposure to the quality improvement programs and changes in associated HAP rates is warranted. On the other hand, the fact that COPD patients did not have a higher prevalence of bronchiectasis, a comorbidity that could be important due to the possibility of bacterial colonization, could also have influenced these results.

Incidence of HAP, (NV)-HAP and VAP during the period 2016–2019 was higher in COPD men than COPD women. When we compare COPD men with COPD women, we observe that men were older, with a higher mean CCI. After matching, no significant differences were found between men and women regarding the use of therapeutic procedures, except the prevalence of undergone surgery which is still significantly higher in men.

Despite improvements in prevention, antimicrobial therapy, and supportive care [30], HAP remains an important cause of morbidity and mortality. HAP has been associated with significant mortality [31,32]. In our study, IHM was similar in COPD patients and in non-COPD patients, with no significant change overtime. Furthermore, sex was not associated with IHM, confirming previous findings suggesting that sex may influence the incidence, but not the severity of these infections [33].

In our cohort, factors associated with IHM during admissions for HAP among men and women with COPD were age, congestive heart failure and cancer/metastatic cancer. It is noteworthy that, among men, those who underwent surgery had lower risk of IHM, a finding previously described by other authors [1]. The reason may be that one of the most important surgical indications is a relatively good baseline health condition [34]. Therefore, the physical condition of these patients may be better than that of patients who cannot undergo general anesthesia, and they are less likely to suffer pneumonia infection because of their suitability for more comprehensive treatment and care, such as sputum aspiration after completion of general anesthesia surgery [35]. 

In the current study, we identified that VAP increased the probability of dying in both men and women with COPD. Previous studies have also reported that COPD is associated with increased mortality rates in VAP patients [36,37]. Several factors could explain the higher rates of mortality in COPD patients with VAP including the adverse influence of COPD on baseline respiratory function, nutritional status of patients with COPD, which may affect adversely the immune response, and previous long-term use of corticosteroids [38,39,40,41,42]. Although our study has not assessed these hypotheses, they might be the focus of a future study. 

The present study has some several limitations. It is retrospective, which makes it difficult to establish a cause–effect relationship. In addition, it relies on a discharge diagnosis codification system. Thus, there is a lack of other relevant clinical data, which might have increased the possibility of information bias and misclassification. Therefore, for example, we have not analyzed data on smoking history that could also affect risk and outcomes of hospital-acquired pneumonia. We have not included this variable because according to the RAE-CMBD methodology in the secondary diagnosis fields (from 2 to 20), should only be recorded those diagnoses that have induced the use of additional therapeutic or diagnosis procedures during the hospital admission or have negatively affected the length of hospital stay (LOHS) or the IHM. In our opinion, smoking history have possibly not affected the clinical course of most patients suffering hospital-acquired pneumonia, and would therefore be under codified providing a false image of the study populations. On the other hand, some important patient characteristics (such as pneumonia severity, degree of airflow obstruction, inflammatory markers, specific type of antibiotic treatment, or use of corticosteroids) could not be analyzed because they were not available in the database. In this way, we did not have data on inhalatory therapy used in COPD patients. This is an important point, particularly for inhaled corticosteroids therapy, since the use of this drugs could be a risk factor for pneumoniae. In addition, not all the inhaled corticosteroids are the same in terms of pneumoniae risk factors [43]. Another important aspect in COPD patients, which we were unable to analyze in our study either, is the use of inhalation drug devices. In particular, the incorrect use could give a greater risk of systemic assimilation of steroids and the appearance of complications [44]. We also did have not data on the long-term antibiotic therapy as preventive in the study patients. Future investigations based on reliable data should include all these data. 

In spite of these limitations, the present study has several strengths. It was carried out at the national level, including both large and small community as well as teaching hospitals. In addition, it includes data from four complete consecutive years, accounting for a potential seasonal variation. Finally, a large database was evaluated, which strengthens our conclusions.

## 5. Conclusions

In summary, our analysis revealed that incidence of HAP was significantly higher in COPD patients than in those without COPD, at the expense of (NV)-HAP but not of VAP. When stratifying by sex, we found that the difference was conditioned by the men. IHM was similar in COPD patients and in non-COPD patients with no significant change overtime. In addition, sex was not associated with IHM, suggesting that sex may influence the incidence but not the prognostic of HAP. It would be essential to provide special attention on prevention of HAP for these patients.

## Figures and Tables

**Table 1:** Incidence, clinical characteristics, and in-hospital outcomes of patients hospitalized with hospital-acquired pneumonia (HAP), in Spain from 2016 to 2019 according to presence of COPD.

		2016	2017	2018	2019	*p*-Value
*n*, (incidence of HAP per 100,000 subjects hospitalized)	COPD	1134 (313)	1181 (300)	1341 (327)	1306 (319)	0.177
	No COPD	**7066 (250)**	**7806 (264)**	**8587 (293)**	**8608 (288)**	<0.001
*n*, (incidence of NV-HAP per 100,000 subjects hospitalized)	COPD	1059 (292)	1085 (276)	1236 (302)	1188 (290)	0.197
	No COPD	**6146 (217)**	**6829 (231)**	**7436 (254)**	**7340 (246)**	<0.001
*n*, (incidence of VAP per 100,000 subjects hospitalized)	COPD	75 (21)	96 (24)	105 (26)	118 (29)	0.157
	No COPD	**920 (33)**	**977 (33)**	**1151 (39)**	**1268 (42)**	<0.001
Age, mean (SD)	COPD	74.74 (10.54)	75.54 (10.46)	75.43 (10.5)	74.97 (10.49)	0.199
	No COPD	**71.26 (13.49)**	**71.79 (13.49)**	**71.94 (13.46)**	**71.45 (13.53)**	0.006
40–64 years old, *n* (%)	COPD	199 (17.55)	167 (14.14)	211 (15.73)	220 (16.85)	0.98
	No COPD	2175 (30.78)	2341 (29.99)	2508 (29.21)	2661 (30.91)	0.970
65–74 years old, *n* (%)	COPD	302 (26.63)	339 (28.7)	365 (27.22)	366 (28.02)	0.705
	No COPD	1636 (23.15)	1744 (22.34)	1998 (23.27)	2044 (23.75)	0.234
75–84 years old, *n* (%)	COPD	424 (37.39)	434 (36.75)	483 (36.02)	469 (35.91)	0.504
	No COPD	**2020 (28.59)**	**2188 (28.03)**	**2362 (27.51)**	**2236 (25.98)**	0.001
≥85 years old, *n* (%)	COPD	209 (18.43)	241 (20.41)	282 (21.03)	251 (19.22)	0.634
	No COPD	**1235 (17.48)**	**1533 (19.64)**	**1719 (20.02)**	**1667 (19.37)**	0.010
CCI index, mean (SD)	COPD	1.6 (1.21)	1.6 (1.14)	1.62 (1.18)	1.7 (1.17)	0.132
	No COPD	**1.44 (1.1)**	**1.46 (1.1)**	**1.51 (1.14)**	**1.52 (1.14)**	<0.001
LOHS, Median (IQR)	COPD	22 (24)	21 (23)	21 (23)	22 (24)	0.641
	No COPD	24 (28)	23 (27)	24 (27)	24 (28)	0.141
IHM, *n* (%)	COPD	338 (29.81)	384 (32.51)	403 (30.05)	395 (30.25)	0.225
	No COPD	2109 (29.85)	2305 (29.53)	2543 (29.61)	2452 (28.49)	0.451

The statistically significant differences are presented in bold type. HAP: Hospital-acquired pneumonia; NV-HAP: non-ventilator hospital-acquired pneumonia; VAP: ventilator-associated pneumonia; COPD: Chronic Obstructive Pulmonary Disease; CCI: Charlson comorbidity index; LOHS: Length of hospital stay; IHM: in-hospital mortality. The prevalence of *Streptococcus pneumoniae* isolation increased significantly from 2016 to 2019 among patients with and without COPD (2.65% and 1.95% vs. 3.37% and 2.75%, respectively).

**Table 2:** Distribution of study covariates and hospital outcomes of WOMEN with and without COPD hospitalized with hospital-acquired pneumonia (HAP) in Spain (2016–2019), before and after propensity score matching (PSM).

	Before PSM			After PSM		
	COPD	No COPD	*p*-Value	COPD	No COPD	*p*-Value
*n*, (incidence of HAP per 100,000 women)	775 (209)	12,038 (202)	0.356	775	775	NA
*n*, (incidence of NV-HAP per 100,000 women)	718 (193)	10,728 (180)	0.058	718	718	NA
*n*, (incidence of VAP per 100,000 women)	57 (15)	1310 (22)	0.007	57	57	NA
Age, mean (SD)	74 (11.97)	74.4 (13.72)	0.422	74 (11.97)	74.20 (13.53)	0.473
40–64 years old, *n* (%)	179 (23.1)	2898 (24.07)	0.537	179 (23.1)	176 (22.71)	0.856
65–74 years old, *n* (%)	**198 (25.55)**	**2358 (19.59)**	**0.001**	**198 (25.55)**	**134 (17.29)**	<0.001
75–84 years old, *n* (%)	234 (30.19)	3442 (28.59)	0.340	234 (30.19)	250 (32.26)	0.380
≥85 years old, *n* (%)	**164 (21.16)**	**3340 (27.75)**	**0.001**	**164 (21.16)**	**215 (27.74)**	0.003
CCI index, mean (SD)	1.42 (1.15)	1.43 (1.1)	0.791	1.42 (1.15)	1.44 (1.12)	0.687
Myocardial infarction, *n* (%)	57 (7.35)	592 (4.92)	0.003	57 (7.35)	59 (7.61)	0.847
Congestive heart failure, *n* (%)	298 (38.45)	3577 (29.71)	<0.001	298 (38.45)	295 (38.06)	0.875
Peripheral vascular disease, *n* (%)	45 (5.81)	578 (4.8)	0.207	45 (5.81)	41 (5.29)	0.657
Cerebrovascular disease, *n* (%)	89 (11.48)	1940 (16.12)	0.001	89 (11.48)	87 (11.23)	0.873
Dementia, *n* (%)	37 (4.77)	839 (6.97)	0.019	37 (4.77)	53 (6.84)	0.082
T2DM, *n* (%)	185 (23.87)	2988 (24.82)	0.552	185 (23.87)	187 (24.13)	0.905
Rheumatoid disease, *n* (%)	25 (3.23)	415 (3.45)	0.743	25 (3.23)	34 (4.39)	0.232
Peptic ulcer, *n* (%)	13 (1.68)	225 (1.87)	0.702	13 (1.68)	22 (2.84)	0.124
Mild Moderate/severe liver disease, *n* (%)	52 (6.71)	807 (6.7)	0.995	52 (6.71)	50 (6.45)	0.838
Hemiplegia or paraplegia, n (%)	30 (3.87)	711 (5.91)	0.019	30 (3.87)	29 (3.74)	0.894
Renal disease, *n* (%)	143 (18.45)	2151 (17.87)	0.681	143 (18.45)	142 (18.32)	0.948
Cancer/Metastatic cancer, *n* (%)	118 (15.23)	2337 (19.41)	0.004	118 (15.23)	115 (14.84)	0.831
AIDS, *n* (%)	7 (0.9)	41 (0.34)	0.013	7 (0.9)	3 (0.39)	0.204
Bronchiectasis, *n* (%)	12 (1.55)	227 (1.89)	0.501	12 (1.55)	17 (2.19)	0.349
Undergone surgery, *n* (%)	**298 (38.45)**	**5389 (44.77)**	**0.001**	**298 (38.45)**	**350 (45.16)**	0.007
Bronchial fibroscopy, *n* (%)	19 (2.45)	264 (2.19)	0.635	19 (2.45)	17 (2.19)	0.736
Computerized axial tomography of thorax, *n* (%)	61 (7.87)	783 (6.5)	0.137	61 (7.87)	66 (8.52)	0.643
Dialysis, *n* (%)	**26 (3.35)**	**609 (5.06)**	**0.034**	**26 (3.35)**	**45 (5.81)**	0.021
Oxygen prior to hospitalization *n* (%)	**92 (11.87)**	**265 (2.2)**	**<0.001**	**92 (11.87)**	**19 (2.45)**	<0.001
LOHS, Median (IQR)	21 (24)	23 (26)	0.016	21 (24)	21 (22)	0.622
IHM, *n* (%)	214 (27.61)	3447 (28.63)	0.542	214 (27.61)	235 (30.32)	0.239

The statistically significant differences are presented in bold type. HAP: Hospital-acquired pneumonia; NV-HAP: non-ventilator hospital-acquired pneumonia; VAP: ventilator-associated pneumonia; T2DM: Type 2 diabetes mellitus; CCI: Charlson comorbidity index; COPD: chronic obstructive pulmonary disease; AIDS: acquired immune deficiency syndrome; LOHS: Length of hospital stay; IHM: in-hospital mortality. NA; Not available.

**Table 3:** Distribution of study covariates and hospital outcomes of MEN with and without COPD hospitalized with hospital-acquired pneumonia (HAP) in Spain (2016–2019), before and after propensity score matching (PSM).

	Before PSM			After PSM		
	COPD	No COPD	*p*-Value	COPD	No COPD	*p*-Value
*n*, (incidence of HAP per 100,000 men)	4187 (368)	20,029 (350)	<0.001	4187	4187	NA
*n*, (incidence of NV-HAP per 100,000 men)	3850 (340)	17,023 (297)	<0.001	3850	3850	NA
*n*, (incidence of VAP per 100,000 men)	337 (28)	3006 (52)	<0.001	337	337	NA
Age, mean (SD)	75.39 (10.19)	69.95 (13.07)	<0.001	75.39 (10.19)	74.95 (11.11)	0.057
40–64 years old, *n* (%)	618 (14.76)	6787 (33.89)	<0.001	618 (14.76)	627 (14.97)	0.782
65–74 years old, *n* (%)	1174 (28.04)	5064 (25.28)	<0.001	1174 (28.04)	1167 (27.87)	0.862
75–84 years old, *n* (%)	1576 (37.64)	5364 (26.78)	<0.001	1576 (37.64)	1565 (37.38)	0.804
≥85 years old, *n* (%)	819 (19.56)	2814 (14.05)	<0.001	819 (19.56)	828 (19.78)	0.810
CCI index, mean (SD)	1.67 (1.17)	1.52 (1.14)	<0.001	1.67 (1.17)	1.67 (1.15)	0.828
Myocardial infarction, *n* (%)	391 (9.34)	2016 (10.07)	0.153	391 (9.34)	375 (8.96)	0.544
Congestive heart failure, *n* (%)	1315 (31.41)	4252 (21.23)	<0.001	1315 (31.41)	1298 (31)	0.688
Peripheral vascular disease, *n* (%)	597 (14.26)	2035 (10.16)	<0.001	597 (14.26)	588 (14.04)	0.778
Cerebrovascular disease, *n* (%)	510 (12.18)	3262 (16.29)	<0.001	510 (12.18)	493 (11.77)	0.567
Dementia, *n* (%)	**162 (3.87)**	**858 (4.28)**	**0.224**	**162 (3.87)**	**226 (5.4)**	0.001
T2DM, *n* (%)	1271 (30.36)	4872 (24.32)	<0.001	1271 (30.36)	1263 (30.16)	0.849
Rheumatoid disease, *n* (%)	63 (1.5)	274 (1.37)	0.492	63 (1.5)	69 (1.65)	0.599
Peptic ulcer, *n* (%)	83 (1.98)	455 (2.27)	0.248	83 (1.98)	97 (2.32)	0.291
Mild Moderate/severe liver disease, *n* (%)	427 (10.2)	2241 (11.19)	0.063	427 (10.2)	419 (10.01)	0.772
Hemiplegia or paraplegia, *n* (%)	173 (4.13)	1307 (6.53)	<0.001	173 (4.13)	152 (3.63)	0.235
Renal disease, n (%)	934 (22.31)	3312 (16.54)	<0.001	934 (22.31)	940 (22.45)	0.875
Cancer/Metastatic cancer, *n* (%)	1050 (25.08)	5362 (26.77)	0.024	1050 (25.08)	1050 (25.08)	0.999
AIDS, *n* (%)	**28 (0.67)**	**170 (0.85)**	**0.239**	**28 (0.67)**	**11 (0.26)**	0.006
Bronchiectasis, *n* (%)	95 (2.27)	408 (2.04)	0.339	95 (2.27)	121 (2.89)	0.073
Undergone surgery, *n* (%)	**1829 (43.68)**	**10,330 (51.58)**	**<0.001**	**1829 (43.68)**	**2022 (48.29)**	<0.001
Bronchial fibroscopy, *n* (%)	109 (2.6)	656 (3.28)	0.024	109 (2.6)	88 (2.1)	0.130
Computerized axial tomography of thorax, *n* (%)	**329 (7.86)**	**1677 (8.37)**	**0.271**	**329 (7.86)**	**398 (9.51)**	0.007
Dialysis, *n* (%)	**230 (5.49)**	**1560 (7.79)**	**<0.001**	**230 (5.49)**	**313 (7.48)**	<0.001
Oxygen prior to hospitalization *n* (%)	**378 (9.03)**	**318 (1.59)**	**<0.001**	**378 (9.03)**	**70 (1.67)**	<0.001
LOHS, Median (IQR)	**22 (23)**	**25 (28)**	**<0.001**	**22 (23)**	**23 (25)**	<0.001
IHM, *n* (%)	1306 (31.19)	5962 (29.77)	0.067	1306 (31.19)	1291 (30.83)	0.723

The statistically significant differences are presented in bold type. HAP: Hospital-acquired pneumonia; NV-HAP: non-ventilator hospital-acquired pneumonia; VAP: ventilator-associated pneumonia; T2DM: Type 2 diabetes mellitus; CCI: Charlson comorbidity index; COPD: chronic obstructive pulmonary disease; AIDS: acquired immune deficiency syndrome; LOHS: Length of hospital stay; IHM: in-hospital mortality. NA. Not available.

**Table 4:** Distribution of study covariates and hospital outcomes of MEN AND WOMEN with COPD hospitalized with hospital-acquired pneumonia (HAP) in Spain (2016–2019), before and after propensity score matching (PSM).

	Before PSM			After PSM		
	COPD Men	COPD Women	*p*-Value	COPD Men	COPD Women	*p*-Value
*n*, (incidence of HAP per 100,000 subjects)	4187 (368)	775 (209)	<0.001	775	775	NA
*n*, (incidence of NV-HAP per 100,000 subjects)	3850 (340)	718 (193)	<0.001	718	718	NA
*n*, (incidence of VAP per 100,000 subjects)	4187 (28)	57 (15)	<0.001	57	57	NA
Age, mean (SD)	75.39 (10.19)	74 (11.97)	<0.001	74.02 (11.95)	74.02 (11.96)	0.999
40–64 years old, *n* (%)	618 (14.76)	179 (23.1)	<0.001	178 (23)	178 (23)	0.999
65–74 years old, *n* (%)	1174 (28.04)	198 (25.55)	0.154	198 (25.58)	198 (25.58)	0.999
75–84 years old, *n* (%)	1576 (37.64)	234 (30.19)	0.001	234 (30.23)	234 (30.23)	0.999
≥85 years old, *n* (%)	819 (19.56)	164 (21.16)	0.304	164 (21.19)	164 (21.19)	0.999
CCI index, mean (SD)	**1.67 (1.17)**	**1.42 (1.15)**	**<0.001**	**1.60 (1.21)**	**1.42 (1.15)**	<0.001
Myocardial infarction, *n* (%)	391 (9.34)	57 (7.35)	0.077	72 (9.3)	57 (7.36)	0.168
Congestive heart failure, *n* (%)	**1315 (31.41)**	**298 (38.45)**	**<0.001**	**240 (31.01)**	**298 (38.5)**	0.002
Peripheral vascular disease, *n* (%)	**597 (14.26)**	**45 (5.81)**	**<0.001**	**115 (14.86)**	**45 (5.81)**	<0.001
Cerebrovascular disease, *n* (%)	510 (12.18)	89 (11.48)	0.584	91 (11.76)	89 (11.5)	0.874
Dementia, *n* (%)	162 (3.87)	37 (4.77)	0.238	27 (3.49)	37 (4.78)	0.202
T2DM, *n* (%)	**1271 (30.36)**	**185 (23.87)**	**<0.001**	**227 (29.33)**	**185 (23.9)**	0.016
Rheumatoid disease, *n* (%)	63 (1.5)	25 (3.23)	0.001	16 (2.07)	25 (3.23)	0.154
Peptic ulcer, *n* (%)	**83 (1.98)**	**13 (1.68)**	**0.571**	**26 (3.36)**	**13 (1.68)**	0.035
Mild Moderate/severe liver disease, *n* (%)	**427 (10.2)**	**52 (6.71)**	**0.003**	**98 (12.66)**	**52 (6.72)**	<0.001
Hemiplegia or paraplegia, *n* (%)	173 (4.13)	30 (3.87)	0.736	39 (5.04)	30 (3.88)	0.268
Renal disease, *n* (%)	934 (22.31)	143 (18.45)	0.017	167 (21.58)	143 (18.48)	0.127
Cancer/Metastatic cancer, *n* (%)	**1050 (25.08)**	**118 (15.23)**	**<0.001**	**188 (24.29)**	**118 (15.25)**	<0.001
AIDS, *n* (%)	28 (0.67)	7 (0.9)	0.474	8 (1.03)	7 (0.9)	0.795
Bronchiectasis, *n* (%)	95 (2.27)	12 (1.55)	0.205	20 (2.58)	12 (1.54)	0.153
Undergone surgery, *n* (%)	**1829 (43.68)**	**298 (38.45)**	**0.007**	**342 (44.19)**	**297 (38.37)**	0.020
Bronchial fibroscopy, *n* (%)	109 (2.6)	19 (2.45)	0.807	19 (2.45)	19 (2.45)	0.999
Computerized axial tomography of thorax, *n* (%)	329 (7.86)	61 (7.87)	0.990	62 (8.01)	61 (7.88)	0.925
Dialysis, *n* (%)	230 (5.49)	26 (3.35)	0.013	37 (4.78)	26 (3.36)	0.157
Oxygen prior to hospitalization *n* (%)	378 (9.03)	92 (11.87)	0.012	73 (9.43)	92 (11.89)	0.118
LOHS, Median (IQR)	22 (23)	21 (24)	0.641	21 (23)	21 (24)	0.563
IHM, *n* (%)	1306 (31.19)	214 (27.61)	0.047	229 (29.59)	214 (27.65)	0.399

The statistically significant differences are presented in bold type. HAP: Hospital-acquired pneumonia; NV-HAP: non-ventilator hospital-acquired pneumonia; VAP: ventilator-associated pneumonia; T2DM: Type 2 diabetes mellitus; CCI: Charlson comorbidity index; COPD: chronic obstructive pulmonary disease; AIDS: acquired immune deficiency syndrome; LOHS: Length of hospital stay; IHM: in-hospital mortality. NA; Not available.

**Table 5:** Multivariable analysis of factors associated with in-hospital mortality during admissions for hospital-acquired pneumonia (HAP), among COPD patients according to sex.

	Men	Women	Both
	OR (95%CI)	OR (95%CI)	OR (95%CI)
40–64 years old	1	1	1
65–74 years old *	**1.29 (1.03–1.62)**	1.05 (0.63–1.76)	1.03 (0.72–1.47)
75–84 years old *	**1.71 (1.37–2.13)**	**2.02 (1.23–3.3)**	**2.06 (1.47–2.87)**
≥85 years old *	**1.81 (1.42–2.32)**	1.6 (0.92–2.77)	**1.79 (1.23–2.6)**
Congestive heart failure *	**1.19 (1.02–1.37)**	**2.2 (1.54–3.15)**	**1.56 (1.22–2)**
Hemiplegia or paraplegia *	**1.66 (1.21–2.3)**	NIFM	NIFM
Cancer/Metastatic cancer *	**1.3 (1.1–1.52)**	**1.78 (1.12–2.84)**	**1.55 (1.16–2.08)**
Undergone surgery *	**0.71 (0.61–0.81)**	NIFM	NIFM
Dialysis *	**3.59 (2.71–4.77)**	**5.3 (2.25–12.45)**	**4.11 (2.4–7.05)**
2017	1.13 (0.93–1.37)	1.11 (0.68–1.82)	1.11 (0.79–1.56)
2018	0.99 (0.82–1.2)	1.04 (0.65–1.65)	0.94 (0.68–1.31)
2019	0.97 (0.8–1.18)	1.04 (0.65–1.66)	0.91 (0.65–1.26)
VAP *	**2.37 (1.85–3.04)**	**3.51 (1.91–6.45)**	**2.58 (1.66–4)**
Men	NA	NA	1.09 (0.87–1.38)

OR Odds Ratio. CI Confidence Interval. * Inidicates those variables significantlly associated with In Hospital Mortality. The statistically significant OR are presented in bold type. NIFM Not included in the final model. NA Not applicable.

## Data Availability

According to the contract signed with the Spanish Ministry of Health and Social Services, which provided access to the databases from the Hospital Discharge Records of the Spanish National Health System (In Spanish: Conjunto Mínimo Basico de Datos (CMBD)), we cannot share the databases with any other investigator, and we have to destroy the databases once the investigation has concluded. Consequently, we cannot upload the databases to any public repository. However, any investigator can apply for access to the databases by filling out the questionnaire available at http://www.msssi.gob.es/estadEstudios/estadisticas/estadisticas/estMinisterio/SolicitudCMBDdocs/Formulario_Peticion_Datos_CMBD.pdf (accessed on 16 March 2021). All other relevant data are included in the paper.

## Supplementary Materials

The following is available online at https://www.mdpi.com/article/10.3390/jcm10225453/s1, Table S1. ICD-10 codes for diagnosis and therapeutic procedures and pressure ulcers used in this investigation. Table S2. Distribution of pneumonia pathogens in patients with and without COPD hospitalized with hospital-acquired pneumonia (HAP) in Spain from 2016 to 2019. Table S3. Distribution of pneumonia pathogens in women and men with COPD hospitalized with hospital-acquired pneumonia (HAP), in Spain (2016–2019), before and after propensity score matching.
