# Supplementary material for: Assessing the Impact of Gender and COPD on the Incidence and Mortality of Hospital-Acquired Pneumonia. A Retrospective Cohort Study Using the Spanish National Discharge Database (2016–2019)
Source: J Clin Med. 2021 Nov 22;10(22):5453. doi: 10.3390/jcm10225453 (PMC8625205; doi:10.3390/jcm10225453)
Supplement: Supplementary file 1 [file jcm-10-05453-s001.zip › jcm-1461250-supplementary.pdf]

Table S1. ICD-10 codes for diagnosis and therapeutic procedures and pressure ulcers used in this investigation

		ICD-10 codes
Hospital acquired pneumonia	Non ventilator hospital-acquired pneumonia (NV-HAP)*	J12 to J18 in any diagnosis fields (2-20) and with a POA indicator coded as "N" who had a hospitalization ≥48 hours
	Ventilator-associated pneumonia (VAP)*	J95.851 in any diagnosis fields (2-20) and a POA indicator coded as "N"
Bronchial fibroscopy		0BJ08ZZ
Computerized axial tomography of thorax		BW24
Dyalisis		5A1D00Z, 5A1D60Z,3E1M39Z
Oxygen prior to hospitalization		Z99.81
Aspergillus		B44.9
Candidiasis		B37.1
Escherichia coli		J15.5
Haemophilus influenzae		J14
Klebsiella pneumoniae		J15
Legionella		A48.1
Non specified *Streptococcus*		J15.4
Other Gram negative bacteria		J15.6
Influenza virus		J09.X1, J10.00 J10.01, J10.08, J11.0, J11.00, J11.08
Other virus		J12.XX
Pseudomonas aeruginosa		J15.1
Staphylococcus aureus		J15.211 AND J15.212
Streptococcus pneumoniae		J13

*Each discharge diagnosis has a "Present on Admission (POA)" indicator assigned according to the ICD-10-CM Official Guidelines for Coding and Reporting (**https://icdlist.com/icd-10/guidelines/**). The reporting options and definitions for POA are "Y" (present at admission); "N" (not present at admission); "U" (lack documentation to determine presence at admission); "W" (provider is unable to clinically determine if the condition was present); and unreported/not used.

Table S2. Distribution of pneumonia pathogens in patients with and without COPD hospitalized with hospital-acquired pneumonia (HAP) in Spain from 2016 to 2019

		2016	2017	2018	2019	p-value
Aspergillus, n(%)	**COPD**	11(0.97)	8(0.68)	7(0.52)	0(0)	0.008
	**No COPD**	17(0.24)	17(0.22)	13(0.15)	29(0.34)	0.093
Candidiasis, n(%)	**COPD**	11(0.97)	12(1.02)	7(0.52)	6(0.46)	0.223
	**No COPD**	49(0.69)	58(0.74)	63(0.73)	80(0.93)	0.315
Escherichia coli, n(%)	**COPD**	28(2.47)	18(1.52)	25(1.86)	21(1.61)	0.321
	**No COPD**	136(1.92)	121(1.55)	151(1.76)	128(1.49)	0.131
Haemophilus influenzae, n(%)	**COPD**	22(1.94)	11(0.93)	23(1.72)	19(1.45)	0.215
	**No COPD**	87(1.23)	91(1.17)	110(1.28)	95(1.1)	0.733
Klebsiella pneumoniae, n(%)	**COPD**	25(2.2)	23(1.95)	33(2.46)	34(2.6)	0.712
	**No COPD**	211(2.99)	203(2.6)	218(2.54)	268(3.11)	0.064
Legionella, n(%)	**COPD**	0(0)	0(0)	2(0.15)	2(0.15)	0.320
	**No COPD**	4(0.06)	5(0.06)	6(0.07)	8(0.09)	0.841
Non specified *Streptococcus*, n(%)	**COPD**	6(0.53)	6(0.51)	1(0.07)	7(0.54)	0.175
	**No COPD**	40(0.57)	21(0.27)	35(0.41)	39(0.45)	0.044
Other Gram-negative bacteria, n(%)	**COPD**	27(2.38)	37(3.13)	33(2.46)	43(3.29)	0.405
	**No COPD**	223(3.16)	245(3.14)	249(2.9)	273(3.17)	0.707
Pseudomonas aeruginosa, n(%)	**COPD**	69(6.08)	84(7.11)	58(4.33)	70(5.36)	0.021
	**No COPD**	317(4.49)	308(3.95)	340(3.96)	342(3.97)	0.271
Streptococcus pneumoniae, n(%)	**COPD**	30(2.65)	30(2.54)	59(4.4)	44(3.37)	0.032
	**No COPD**	138(1.95)	185(2.37)	215(2.5)	237(2.75)	0.012
Staphylococcus aureus, n(%)	**COPD**	30(2.65)	46(3.9)	36(2.68)	38(2.91)	0.241
	**No COPD**	180(2.55)	210(2.69)	250(2.91)	247(2.87)	0.486
Influenza virus, n(%)	**COPD**	12(1.06)	12(1.02)	19(1.42)	10(0.77)	0.439
	**No COPD**	84(1.19)	51(0.65)	136(1.58)	118(1.37)	<0.001
Other virus, n (%)	**COPD**	5(0.44)	5(0.42)	4(0.3)	12(0.92)	0.133
	**No COPD**	36(0.51)	41(0.53)	63(0.73)	62(0.72)	0.210

T2DM: Type 2 diabetes mellitus;

Table S3. Distribution of pneumonia pathogens in women and men with COPD hospitalized with hospital-acquired pneumonia (HAP), in Spain (2016-19), before and after propensity score matching.

	**Before PSM**			**After PSM**		
	**COPD Men**	**COPD Women**	**p-value**	**COPD Men**	**COPD Women**	**p-value**
Aspergillus, n(%)	17(0.41)	9(1.16)	0.007	1(0.13)	9(1.16)	0.011
Candidiasis, n(%)	32(0.76)	4(0.52)	0.455	2(0.26)	4(0.52)	0.413
Escherichia coli, n(%)	77(1.84)	15(1.94)	0.855	9(1.16)	15(1.94)	0.217
Haemophilus influenzae, n(%)	61(1.46)	14(1.81)	0.464	11(1.42)	13(1.68)	0.681
Klebsiella pneumoniae, n(%)	104(2.48)	11(1.42)	0.070	21(2.71)	11(1.42)	0.074
Legionella, n(%)	4(0.1)	0(0)	0.389	1(0.13)	0(0)	0.317
Non specified *Streptococcus*, n(%)	18(0.43)	2(0.26)	0.488	4(0.52)	2(0.26)	0.413
Other Gram-negative bacteria, n(%)	124(2.96)	16(2.06)	0.166	27(3.49)	16(2.07)	0.089
Pseudomonas aeruginosa, n(%)	246(5.88)	35(4.52)	0.133	41(5.3)	35(4.52)	0.480
Streptococcus pneumoniae, n(%)	132(3.15)	31(4)	0.224	23(2.97)	30(3.88)	0.328
Staphylococcus aureus, n(%)	131(3.13)	19(2.45)	0.312	21(2.71)	19(2.45)	0.749
Influenza virus, n(%)	44(1.05)	9(1.16)	0.784	6(0.78)	9(1.16)	0.436
Other virus, n(%)	20(0.48)	6(0.77)	0.294	4(0.52)	6(0.78)	0.526
